# Development of Artificial Intelligence for Determining Major Depressive Disorder Based on Resting-State EEG and Single-Pulse Transcranial Magnetic Stimulation-Evoked EEG Indices

**DOI:** 10.3390/jpm14010101

**Published:** 2024-01-17

**Authors:** Yoshihiro Noda, Kento Sakaue, Masataka Wada, Mayuko Takano, Shinichiro Nakajima

**Affiliations:** 1 Department of Neuropsychiatry, Keio University School of Medicine, Tokyo 160-8582, Japan; 2 Division of DX Promotion, Teijin Limited, Tokyo 100-8585, Japan; 3 Teijin Pharma Limited, Tokyo 100-8585, Japan

**Keywords:** artificial intelligence (AI), machine learning (ML), major depressive disorder (MDD), neurophysiology, transcranial magnetic stimulation (TMS), TMS-EEG

## Abstract

Depression is the disorder with the greatest socioeconomic burdens. Its diagnosis is still based on an operational diagnosis derived from symptoms, and no objective diagnostic indicators exist. Thus, the present study aimed to develop an artificial intelligence (AI) model to aid in the diagnosis of depression from electroencephalography (EEG) data by applying machine learning to resting-state EEG and transcranial magnetic stimulation (TMS)-evoked EEG acquired from patients with depression and healthy controls. Resting-state EEG and single-pulse TMS-EEG were acquired from 60 patients and 60 healthy controls. Power spectrum analysis, phase synchronization analysis, and phase-amplitude coupling analysis were conducted on EEG data to extract feature candidates to apply different types of machine learning algorithms. Furthermore, to address the limitation of the sample size, dimensionality reduction was performed in a manner to increase the quality of information by featuring robust neurophysiological metrics that showed significant differences between the two groups. Then, nine different machine learning models were applied to the data. For the EEG data, we created models combining four modalities, including (1) resting-state EEG, (2) pre-stimulus TMS-EEG, (3) post-stimulus TMS-EEG, and (4) differences between pre- and post-stimulus TMS-EEG, and evaluated their performance. We found that the best estimation performance (a mean area under the curve of 0.922) was obtained using receiver operating characteristic curve analysis when linear discriminant analysis (LDA) was applied to the combination of the four feature sets. This study showed that by using TMS-EEG neurophysiological indices as features, it is possible to develop a depression decision-support AI algorithm that exhibits high discrimination accuracy.

## 1. Introduction

Major depressive disorder (MDD) is a common psychiatric disorder, with a lifetime prevalence of approximately 5–17% [1]. MDD was ranked by World Health Organization as the third leading cause of the global burden of disease in 2008 and is projected to become the first leading cause by 2030 [2]. MDD is a clinical diagnosis and is made primarily by interviewing the patient’s medical history and assessing mental status. The interview includes a medical history; family history; social history, including education and employment history, preferences, hobbies and habits; and drug use history along with psychiatric symptoms [3]. There, ancillary and collateral information from the patient’s family and friends is often a crucial part of the psychiatric evaluation.

MDD not only causes severe functional disability but also adversely affects interpersonal relationships, thus reducing the quality of life. The risk of suicide is also increased in patients with MDD [4]. Furthermore, approximately 30% of patients with MDD do not respond to conventional medication and present with treatment-resistant depression (TRD). Patients with TRD have been shown to have a higher prevalence of psychiatric comorbidities, twice the use of outpatient medical resources, three times the number of hospital days, and a 23% higher all-cause mortality rate compared to those with non-TRD [5,6]. Early identification of high-risk patients with TRD is crucial for the efficient distribution of health care resources, especially since TRD imposes significant personal and social burdens on patients [7].

The etiology of MDD is presumed to be multifactorial, ranging from biological factors, notably genetic factors [8], to environmental factors, including psychosocial factors. Recent research on depression, however, suggests that more complex neuromodulatory systems and neural networks are involved in its pathogenesis, possibly secondary to disturbances in neurotransmitter systems, such as gamma-Aminobutyric acid (GABA), an inhibitory neurotransmitter, and glutamate, an excitatory neurotransmitter [9]. However, currently, no objective biomarkers have yet been established to aid in the diagnosis of depression. In recent years, simultaneous transcranial magnetic stimulation (TMS)-electroencephalography (EEG) measurement, which can directly measure neurophysiological functions of the human cerebral cortex noninvasively, has been applied to MDD and other psychiatric disorders and may contribute to the elucidation of the pathophysiological basis of depression [10].

On the other hand, EEG-based machine learning (ML) models for MDD discrimination have attracted increasing attention in recent years. Although it is possible to objectively distinguish between MDD and healthy controls using EEG data, the high complexity of EEG data and the low accuracy of the detection models still limit their clinical applications. While there are several representative previous studies in this area, Loh et al. proposed a new convolutional neural network (CNN)-based deep learning model for automatically discriminating patients with MDD from healthy controls (HCs). In the study, a short-time Fourier transform was first applied to EEG signals to obtain spectrogram images of patients with MDD and HC. These spectrogram images were fed to the CNN model, which automatically distinguished patients with MDD from HCs. The EEG signals used in this study were obtained from a public database and included 34 patients with MDD and 30 HCs. The best classification accuracy, precision, sensitivity, and specificity obtained with the hold-out validation were 99.6%, 99.4%, 99.7%, and 99.5%, respectively. Although the detection model was very accurate in determining MDD, they concluded that it needs to be validated in a more diverse MDD database before it can be applied clinically [11]. Furthermore, Tasci et al. used a novel lattice-based feature extraction model called Twin Pascal’s Triangles Lattice Pattern to generate dynamic patterns from EEG signals, which were combined with statistical features and fed into a k-nearest neighbor (KNN) classifier. They validated their model on a public dataset consisting of 24 patients with MDD and 29 HCs. Their proposed model achieved a binary classification accuracy of 83.96% with leave-one-subject-out cross-validation and 100% with 10-fold cross-validation strategies. This accuracy was higher than that obtained by other studies using the same data set [12]. In addition, Aydemir et al. proposed a new automatic MDD detection system using EEG signals that consisted of three steps: multi-step feature generation based on melamine patterns and discrete wavelet transform, selection of the most relevant features using nearest neighbor component analysis, and support vector machine (SVM) and KNN classification. The study applied the melamine pattern, which uses the molecular structure of melamine to generate 1536 features. With respect to the detection of MDD, studies using voice and facial biomarkers failed to achieve high detection accuracy, while studies using EEG signals showed the possibility of combining nonlinear feature extraction and machine learning techniques to achieve high detection performance [13].

Furthermore, recent notable AI studies using EEG data related to depression treatment include the following works. One study by Wu and colleagues used a machine learning algorithm designed for resting-state EEG to identify a neurobiological signature that could predict a patient’s response to antidepressant treatment. They found that symptom improvement was strongly predicted by this EEG signature, which was specific to sertraline and could be generalized across different study sites and EEG equipment. This signature also reflected general antidepressant medication responsivity and was related to the outcome of a TMS treatment. In addition, the sertraline resting-state EEG signature was found to index prefrontal neural responsivity. They concluded that these findings could potentially lead to more personalized treatment approaches for depression [14]. The other study conducted by Zhang and colleagues used machine learning to identify two distinct subtypes of post-traumatic stress disorder (PTSD) and major depressive disorder (MDD). These subtypes were identified based on different patterns of functional connectivity within the brain, specifically within the frontoparietal control network and the default mode network. They analyzed data from four datasets of patients with PTSD and MDD using high-density resting-state EEG. They found that these subtypes could be identified across different datasets, suggesting that their findings were generalizable. Moreover, the subtype that differed most from healthy controls in terms of functional connectivity was less responsive to psychotherapy for PTSD and did not respond to antidepressant medication for MDD. However, both subtypes responded well to two different forms of repetitive transcranial magnetic stimulation therapy for MDD [15].

In view of the above background, we developed a model for MDD detection using TMS-EEG data with single-pulse TMS in the left dorsolateral prefrontal cortex (DLPFC), which is considered to be dysfunctional in MDD, in addition to resting-state EEG data. In this study, we specifically calculated changes in cortical reactivity and connectivity during TMS using high-density EEG and compared group differences between the MDD and HC groups to identify neurophysiological indices specific to MDD, which were used as important features in the present study. Furthermore, the present study sought to identify not only a single neurophysiological index in resting EEG or TMS-evoked EEG with single-pulse TMS, but also a combination of these modalities and features that could be a feature pattern to help differentiate MDD from HCs. Moreover, we specifically aimed to identify the feature variables of high importance by featuring each EEG index in the frontal region that is more closely related to the pathophysiology of psychiatric disorders. We also sought to create an artificial intelligence (AI) model to determine depression employing these features and to evaluate the feasibility of implementing this model.

## 2. Materials and Methods

### 2.1. Participants

The selection criteria for patients with MDD and HCs were as follows. *Inclusion criteria for the HC group:* Participants (1) who were between 18 and 65 years of age at the time of obtaining consent; (2) no history of neuropsychiatric disorders at screening assessed by board-certified psychiatrists; (3) normal cognitive function assessed by a Mini-Mental State Examination score of at least 27 points; and (4) not receiving any prescriptions for central nervous system agonists, including psychotropic medications. *Inclusion criteria for patients with MDD:* Participants (1) who were between 18 and 65 years of age at the time of obtaining consent; (2) who are deemed by the investigator to be capable of obtaining written consent; (3) who are outpatients of the Department of Neuropsychiatry at Keio University Hospital; and (4) who meet the diagnostic criteria for MDD in the Diagnostic Standards of Mental Disorders (DSM-5) of the American Psychiatric Association. *Exclusion criteria for all participants:* Participants (1) with cerebral organic diseases (e.g., intracranial organic lesions of moderate severity or higher, neurodegenerative diseases, etc.); (2) with a history of convulsive seizures or epilepsy; (3) with substance-related disorders in the past 6 months; (4) with serious or unstable physical illness; (5) with obvious hearing impairment; (6) who have received electro-convulsive therapy (ECT) treatment within the past 6 months; (7) with contraindications to TMS or MRI, such as magnetic metal implants, pacemakers, claustrophobia, etc.; and (8) whose head, neck, or body size is not suitable for the magnetic resonance imaging (MRI) scanner.

Note that the study was conducted in accordance with the Declaration of Helsinki, and the protocol (ID: 20170152) was reviewed and approved by the Ethics Committee of Keio University School of Medicine. Written informed consent was obtained from each participant in the study. In this study, EEG data from 60 patients with MDD and 60 HCs were used. Of note, Although both groups were matched for sex to a degree, age could not be matched completely. Thus, age was controlled for a posteriori when statistically comparing data between the MDD and HC groups. In addition, all patients with MDD in the present study had controlled medications ranging from 150 mg/day to 225 mg/day of venlafaxine exclusively.

### 2.2. Resting-State EEG and TMS-EEG Measurement Method

Resting-state EEG and TMS-EEG were measured using a TMS-compatible 64-channel EEG system and an EEG cap with silver C-ring slit electrodes (TruScan LT: DEYMED Diagnostic Ltd., Hronov, Czech Republic). All electrodes were referenced to an electrode connected to the right earlobe, and the ground electrode was placed on the left earlobe. EEG signals were recorded at a sampling rate of 3 kHz, and the impedance between the scalp and electrodes was maintained below 5 kΩ throughout the experiments. Resting-state EEG data were acquired for 5 min with eyes closed and awake, and simultaneous TMS-EEG measurements with single-pulse TMS were performed by applying 80 pulses to the left dorsolateral prefrontal cortex (DLPFC). For the TMS-EEG, a figure-8 coil (DuoMAG 70BF, DEYMED Diagnostic s.r.o., Hronov, Czech Republic) with a diameter of 70 mm × 2 windings was used. Identification of the dorsolateral prefrontal cortex (DLPFC), the target site of TMS, was performed in all participants using MRI-guided neuronavigation (Brainsight, Rogue Research Inc., Montréal, QC, Canada). Here, the TMS coil was placed on the scalp at MNI coordinates [x = −38, y = 44, z = 26] at a 45 degree angle to the midline. Other details of the TMS-EEG experimental procedures have been reported in our previously published articles [16,17].

### 2.3. EEG Data Analysis

To develop a discriminator to aid in the diagnosis of MDD and HC, we first performed power spectrum analysis, phase synchronization analysis, and phase-amplitude coupling analysis on the EEG data to prepare for feature extraction and model design. Specifically, the EEG data were first denoised using ICA. Then, the processed EEG data were converted to features; thereafter, feature selection was performed.

#### 2.3.1. EEG Data Preprocessing

First, resting-state EEG was epoched in a non-overlapping 6 s interval [18], and the TMS-EEG was epoched in a 4 s interval from 2 s before to 2 s after TMS. For the TMS-EEG data, EEG data were baseline corrected at this stage. Next, the electrode channels with continuously contaminated large noise, including contact problems, and the epochs with large noise were automatically removed based on preset threshold values. Then, the first ICA was applied to the TMS-EEG data to remove TMS decay artifacts followed by bandpass filtering in the 0.5–100 Hz bandwidth as well as 50 Hz power line noise notch filtering (48–52 Hz). Subsequently, the TMS-EEG data were down-sampled from 3 kHz to 1 kHz here. Then, the second ICA was applied to the preprocessed EEG data for further potential noise rejection [19]. Of note, for the TMS-EEG data, the number of ICs removed by the first and second ICA data cleaning was kept within 20% of the total for both processes combined (i.e., within 12 ICs). On the other hand, for resting-state EEG data, the number of ICs removed by ICA cleaning was less than 8% of the total.

The preprocessed EEG data were averaged over all epochs. Then, the pre-stimulus TMS-EEG data (−1550 ms to −50 ms) and post-stimulus TMS-EEG data (50 ms to 550 ms) were extracted. In addition, to eliminate the possibility of potential noise contamination, such as physical TMS decay and TMS-evoked muscle activity, as much as possible, the TMS-EEG data from −50 ms to 50 ms were not used in this study. For the frequency analysis of the preprocessed TMS-EEG data, the window function was set to include at least two cycles of 4 Hz in the θ band.

#### 2.3.2. Power Spectrum Analysis

For the power spectrum analysis, the calculation was confined to 17 electrodes in the frontal region, and average values were computed for each of the θ (4–7 Hz), α (8–13 Hz), β (14–30 Hz), and γ (30–45 Hz) bands using the open source software EEGLAB toolbox [20] running on MATLAB software (R2020a, the MathWorks Inc., Natick, MA, USA).

#### 2.3.3. Phase Synchronization Analysis with wPLI

Phase synchronization between each channel at 17 electrode sites in the frontal region was calculated using the weighted phase lag index (wPLI) [21,22,23] and averaged for each of the θ, α, β, and γ bands using the open source software FieldTrip toolbox (20221223) [24].

#### 2.3.4. Phase-Amplitude Coupling Analysis

Phase-amplitude coupling analysis [25] in the left DLPFC region, corresponding to F3, F5, and AF3 electrode sites, was performed as follows. First, the frequency bands of interest were filtered. Then, the amplitude and phase components were extracted using the Hilbert transform. Subsequently, based on those values, phase-amplitude coupling was calculated, and the strength of the coupling was indexed with the modulation index (MI). In this study, we specifically calculated the average θ-phase and γ-amplitude coupling and α-phase and γ-amplitude coupling with reference to the comodulograms created by the mean MI values using the open source software Brainstorm toolbox (7-Nov-2018) [26].

### 2.4. EEG Feature Extraction

The preprocessed resting-state EEG, pre-stimulus TMS-EEG, and post-stimulus TMS-EEG were converted into the following three types of features: (1) for resting-state EEG, features were computed for each epoch, and the average of the values for all epochs was calculated (the acronym for these features was named RST); (2) for the pre- and post-stimulus TMS-EEG, the features of the signal after additive averaging were calculated (the acronyms of these features were named PRE and PST, respectively); and, (3) the difference between the pre- and post-stimulus TMS-EEG signals was calculated to obtain the features of pre- and post-stimulus TMS-EEG changes (the acronym for these features was named DIF).

**Figure 1:**
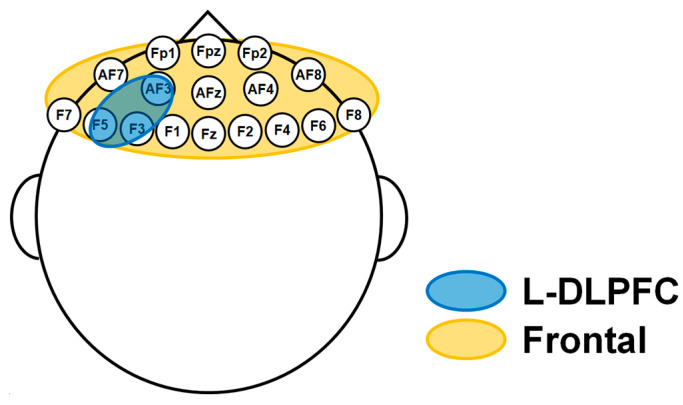
The regions of interest analyzed in this study.

In this study, the analysis was confined to 17 electrodes located in the frontal region, which is more closely related to the pathophysiology of psychiatric disorders (see Figure 1). Furthermore, since machine learning with a small number of data sets generally has the potential for overtraining and poor performance of the model, this study employed a more aggressive dimensionality reduction to overcome this problem and transformed the data into physiologically more informative features [27]. Specifically, in this study, we selected as features only those parameters that differed significantly between the two groups (MDD vs. HC) at the *p* < 0.01 level using the Mann–Whitney U test [28] for the neurophysiological indexes from the potential candidates for features.

In the present study, we focused the analysis on the frontal region, which is assumed to be more closely related to the pathophysiology of depression and other psychiatric disorders.

### 2.5. Dataset Selection, Machine Learning, and Model Performance Evaluation

Using the results of analyses of resting-state EEG data and TMS-EEG data from patients with MDD and HCs, we applied machine learning to create a model to discriminate between the two groups as follows. Specifically, we created feature sets using (1) resting-state EEG only, (2) TMS-EEG only, and (3) combinations of these modalities. We applied machine learning to these feature sets to create a model that can discriminate whether the participant has depression or not based on EEG data.

**Figure 2:**
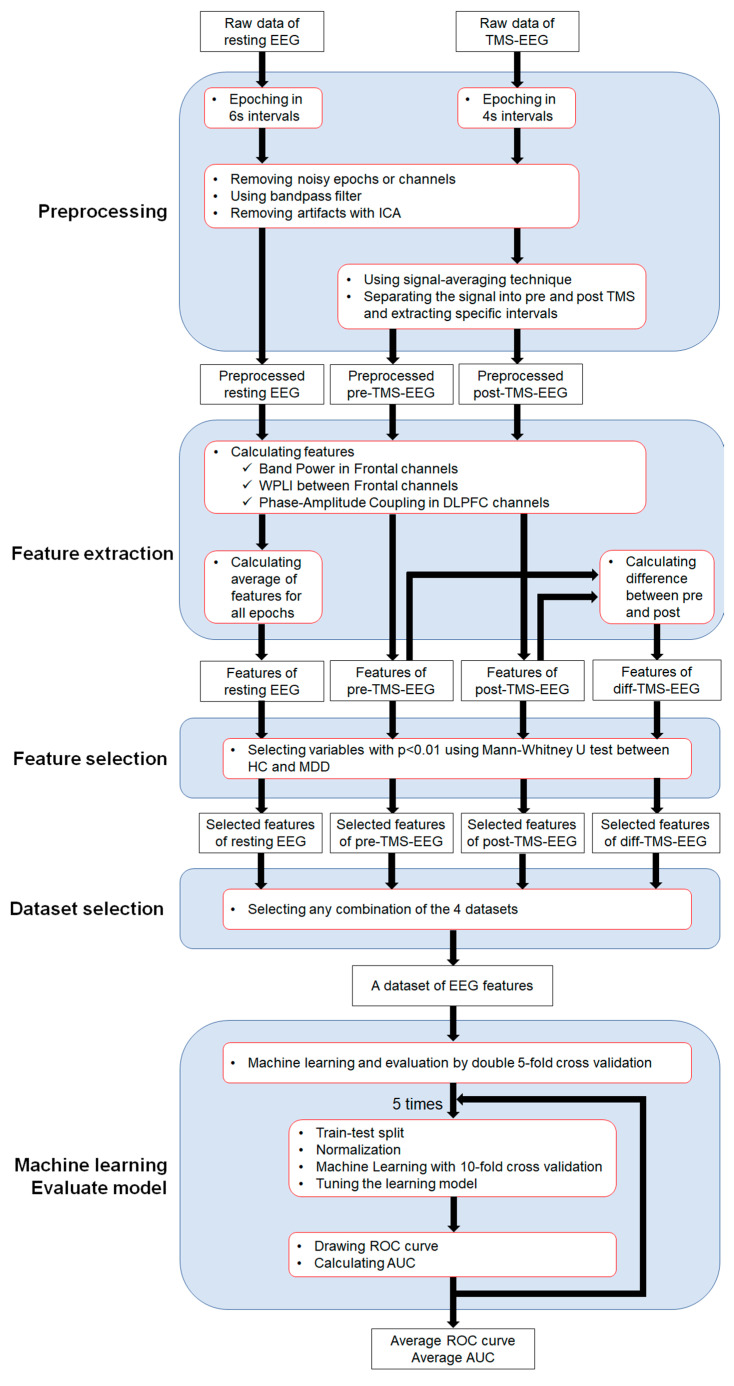
The flow of the analysis procedure in this study. The EEG data analysis in the present study consists of the following five components: (1) Preprocessing, (2) Feature extraction, (3) Feature selection, (4) Dataset selection, and (5) Machine learning and model evaluation.

Due to the small sample size of 120 cases in the present study, we used five-fold outer loop double cross-validation [29,30] to evaluate the estimation performance of the model. Furthermore, in the present study, nine different machine learning models were used to compare the differences in estimation performance for each of the models used as well as the differences in estimation performance for the different combinations of features used. The flow of the analysis procedure is shown in Figure 2. Specifically, we applied 9 different machine learning models (logistic regression (LR), linear discriminant analysis (LDA), SVM, KNN, naïve bayes (NB), decision tree (DT), random forest (RF), extra trees (ET), and lightGBM (LG)) to all combinations generated from the four feature datasets (resting-state EEG (RST), pre-stimulus TMS-EEG (PRE), post-stimulus TMS-EEG (PST), and differences between pre- and post-stimulus TMS-EEG (DIF)) and created each model based on these dataset.

For nested machine learning, the data were standardized, and each dataset was split into training data and test data. Then, machine learning in each model was performed using 10-fold cross-validation. We then performed hyperparameter tuning with a random grid search [31] and applied the tuned model to the test data to calculate the receiver operating characteristic (ROC) curves and area under the curves (AUCs), which indicate model performance. Here, we calculated the five ROC curves and AUCs by 10-fold double cross-validation, and the average ROC curves and average AUCs were calculated using the average method [32]. Note that machine learning and model performance evaluation using ordinal and the outer loop of double cross-validation were performed using PyCaret [33] and scikit-learn in Python.

In addition, for each combination of feature sets for which the model performed well, we also calculated the mean permutation importance [34] for the corresponding trained models.

### 2.6. Statistical Analysis

One-way analysis of covariance with age as a covariate was performed for neurophysiological findings in the EEG that could discriminate between HC and MDD. In addition, we also explored the relationship between the identified critical EEG features and clinical (MADRS score and State-Trait Anxiety Inventory (STAI) score) or cognitive (MMSE score) outcomes with Spearman’s rank correlation coefficient to determine the underlying neurophysiological and clinical implications for both groups (HC and MDD) in this study. A significance level of 0.05 was set for these analyses due to the nature of an exploratory investigation.

## 3. Results

### 3.1. Clinico-Demographic Information

Participants’ demographic data in this study are summarized in Table 1.

### 3.2. Features Extracted from EEG Data

**Figure 3:**
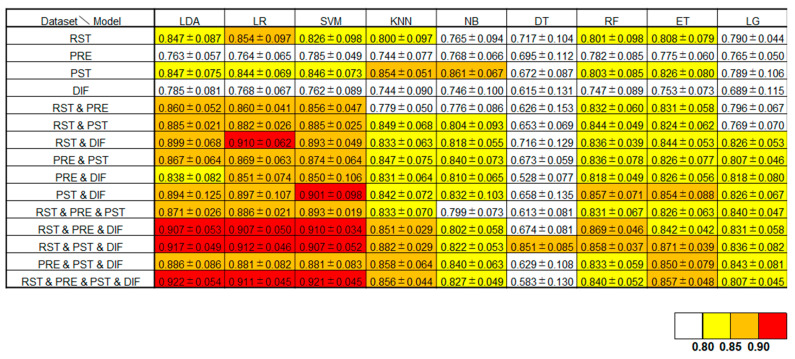
A summary of the mean AUC and its standard deviation, which is the estimated performance of each model created using nine different machine learning methods.

The mean AUCs and their standard deviations (S.D.), which are the estimated performance of each model created with nine different machine learning models for all feature set combinations, are shown in Figure 3. The best estimation performance was obtained when LDA was applied to the machine learning model by combining the feature sets of the four modalities of RST, PRE, PST, and DIF with a mean AUC (±S.D.) of 0.922 ± 0.054. 

For all feature combinations, the mean AUC and its standard deviation, which is the estimated performance of each model created using nine different machine learning methods, are shown in this panel.

### 3.3. MDD Discriminant Analyses Using Various ML Methods

**Figure 4:**
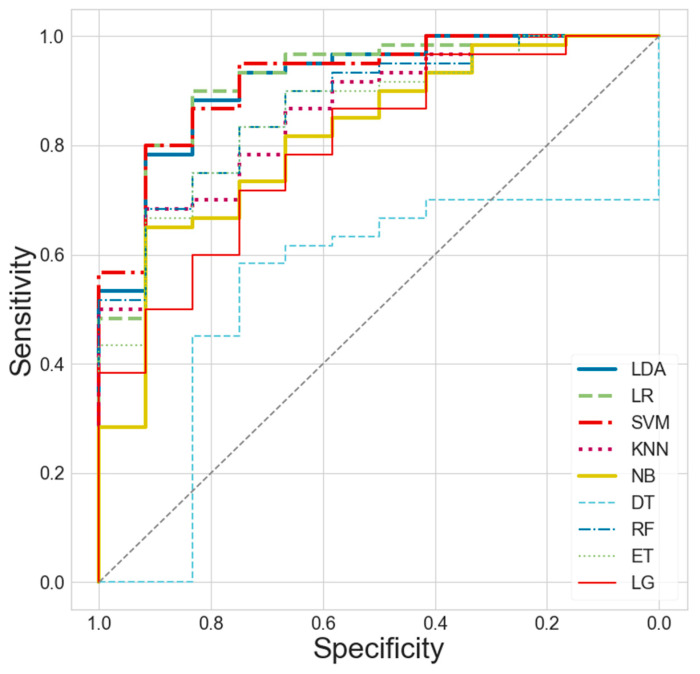
Comparisons of the ROC (receiver operating characteristic) curves of different machine learning models when combining all four modality feature sets of RST, PRE, PST, and DIF.

The ROC curves for different machine learning models were compared when all feature sets from the four modalities were combined. The results showed that LR and SVM, headed by LDA, performed the best, followed by KNN, RF, and ET and then NB and LG, with DT having the lowest performance, in that order (see Figure 4).

The ROC curves of the various machine learning models were compared when combining the feature sets of all four modalities. The results showed that LR and SVM, led by LDA, had the best performance, followed by KNN, RF, ET, NB, and LG in this order, with DT having the lowest performance.

**Figure 5:**
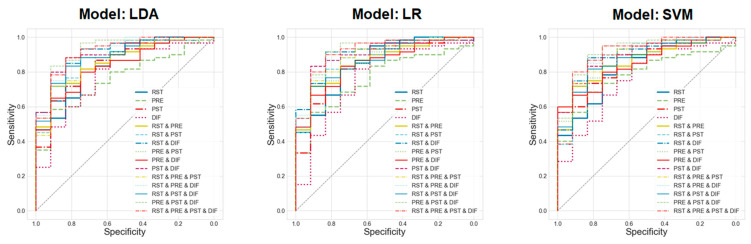
Comparison of the ROC curves for different combinations of four feature sets (RST, PRE, PST, and DIF) when LDA, LR, and SVM were applied as machine learning models.

Next, we compared the mean ROC curves for different combinations of feature sets of the four modalities when applying the top three machine learning models (i.e., LDA, LR, and SVM) that performed very well (see Figure 5). For all models, it was found that the higher the number of feature sets combined, the better the performance.

The mean ROC curves for different combinations of features for the four modalities were compared when the top three machine learning models (i.e., LDA, LR, and SVM) were applied. The results showed that for all models, the higher the number of feature sets combined, the better the performance.

**Figure 6:**
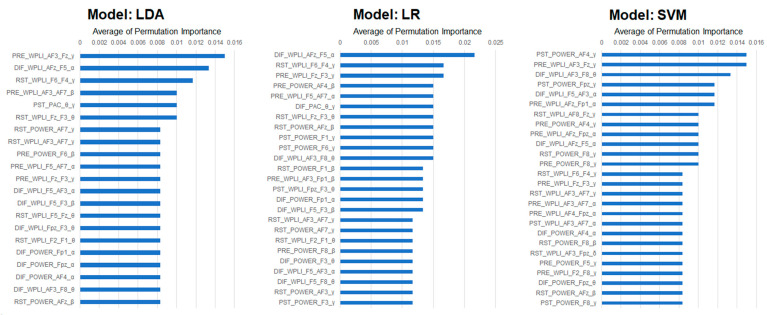
The top 20 mean permutation importance variables of each model when LDA, LR, and SVM were applied as machine learning models using all four feature set combinations.

The mean permutation importance variables [34] of the model when LDA, LR, and SVM were applied as machine learning models using all four feature combinations that performed well in this study yielded the following results (see Figure 6).

The mean permutation importance variables of the models when LDA, LR, and SVM were applied as machine learning models are shown in the graphics with all four feature combinations that performed well in this study.

Furthermore, there were five permutation importance variables that commonly emerged in all of the top three machine learning models (LDA, LR, and SVM): (1) RST_power_AFz_beta; (2) RST_wPLI_F6-F4_gamma; (3) DIF_wPLI_F5-AF3_alpha; (4) DIF_wPLI_AFz-F5_alpha; and (5) DIF_wPLI_AF3-F8_theta (see Table 2). 

### 3.4. EEG Neurophysiological Findings That May Contribute to MDD Discrimination

**Figure 7:**
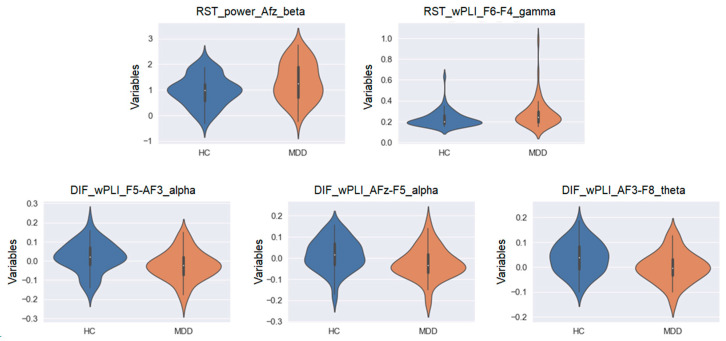
The violin plots of the mean permutation importance variables showing significant group differences between the HC and MDD groups.

Here, the MDD group specifically showed significantly increased beta power at the AFz electrode (F_1,117_ = 7.331, *p* = 0.008) and wPLI gamma phase synchronization between the F6 and F4 electrodes (F_1,117_ = 4.688, *p* = 0.032) in the resting-state EEG compared to the HC group. On the other hand, the MDD group showed significantly lower changes in wPLI alpha phase synchronization between F5 and AF3 electrodes (F_1,117_ = 8.444, *p* = 0.004), wPLI alpha phase synchronization between AFz and F5 electrodes (F_1,117_ = 7.334, *p* = 0.008), and wPLI theta phase synchronization between AF3 and F8 electrodes (F_1,117_ = 9.167, *p* = 0.003) pre- and post-TMS than the HC group (see Figure 7). 

Compared to the HC group, the MDD group showed significantly increased beta power at the AFz electrode and wPLI gamma phase synchronization between F6 and F4 electrodes in the resting-state EEG. On the other hand, compared to the HC group, the MDD group showed significantly decreased wPLI alpha phase synchronization between F5 and AF3 electrodes, wPLI alpha phase synchronization between AFz and F5 electrodes, and wPLI theta phase synchronization between AF3 and F8 electrodes before and after TMS.

### 3.5. Clinical and Cognitive Correlations with the Identified Critical EEG Features

In the present study, we performed correlation analyses between clinical and cognitive measures (MADRS scores, STAI scores, and MMES scores) and the identified critical EEG features. As a result, we found significant correlations between beta power at the AFz electrode in the resting-state EEG and the MADRS score (rho = 0.28, *p* = 0.029, n = 60) as well as the STAI scores (trait: rho = 0.36, *p* = 0.010, n = 60; state: rho = 0.37, *p* = 0.0078, n = 60) in the MDD group. On the other hand, however, no such correlations were observed in the HC group.

## 4. Discussion

In this study, the best estimation performance was achieved when all four feature sets (RST, PRE, PST, and DIF) were combined and LDA was applied as the machine learning model. This integration model yielded a mean AUC of 0.922. When comparing the ROC curves for various machine learning models with all the combined feature sets, the best performance was observed with LDA followed by LR and SVM. Furthermore, comparisons of the mean ROC curves for different combinations of the four feature sets revealed an increase in model performance with the inclusion of a higher number of feature sets.

Previous research applying machine learning to EEG data has often fallen short due to the lack of appropriate model validations within the studies [35]. For example, many studies have overestimated the performance of the created models by evaluating their performance without preparing test data with over-trained models or by showcasing only the coincidental best accuracy obtained with the best combination of datasets [18]. In contrast, this study employed the outer loop of five-fold double cross-validation to evaluate the estimation performance of the model with the calculation of the mean AUC, thereby avoiding overestimation or underestimation of the model’s performance. The outer loop of double cross-validation, in this context, means that cross-validation is nested and performed twice. Since this approach measures the mean performance while comprehensively specifying the test data in the dataset, it can accurately evaluate the estimation performance of the model when the sample size is small. Thus, in this study, we applied the outer loop of five-fold double cross-validation based on previous research [18] in order to reduce variations in AUCs and achieve appropriate variable selection.

By calculating the mean permutation importance in the models derived from applying LDA, LR, and SVM in this study, five variables emerged as common across all models. Specifically, compared to the HC group, a significant increase was observed in the beta power at the AFz electrode and wPLI gamma phase synchronization between the F6 and F4 electrodes in the resting-state EEG for the MDD group. Conversely, wPLI alpha phase synchronization between the F5 and AF3 electrodes as well as between the AFz and F5 electrodes and wPLI theta phase synchronization between the AF3 and F8 electrodes before and after TMS were decreased. Thus, when compared to the HC group, the MDD group demonstrated increased beta power and gamma phase synchronization in the right prefrontal cortex during the resting state and decreased alpha and theta phase synchronization in the left prefrontal area before and after TMS. These findings may serve as important components of the AI discrimination model. Furthermore, the results of the relationship between depressive symptoms using the MADRS, anxiety symptoms using the STAI, and cognitive function using the MMSE and the identified critical EEG features showed that the enhancement of prefrontal central beta power at resting state was significantly correlated with the severity of depressive and anxiety symptoms in the MDD group. However, no such relationships were found in the HC group.

Given that previous studies have indicated that the right prefrontal cortex in MDD is relatively hyperexcited [36,37], the increased gamma phase synchronization between F6 and F4 electrodes may reflect this endophenotype. Moreover, since beta oscillations [38] represent endophenotypes reflecting GABA-A receptor function and gamma oscillations reflecting glutamate N-methyl-D-aspartate receptor function as well as the excitatory and inhibitory balance, it is conceivable that these neurophysiological functions in the prefrontal cortex could be compensatorily and pathologically enhanced in the MDD group compared to the HC group. Furthermore, as alpha and theta oscillations [39,40,41,42] constitute the basic rhythms in the healthy condition, neurophysiological functions associated with alpha and theta oscillations immediately after TMS could be relatively reduced in the MDD group compared to the HC group. Thus, the results of mean permutation importance, which were common to all of the top three models demonstrating superior estimation performance in identifying depression in this study, can be reasonably interpreted within the context of the neurobiological basis and pathophysiological mechanisms of depression. Consequently, it is suggested that the model may be distinguishing depression based on features of neurophysiological significance. Furthermore, when these neurophysiological findings are interpreted in the context of depression, increased beta power and increased synchronization of gamma oscillations in the right prefrontal cortex region at resting state may reflect findings of anxiety and agitation due to relative overexcitation in this region. In fact, TMS therapy for depression often applies inhibitory neuromodulation to the right DLPFC. In contrast, synchronization of theta and alpha oscillations in the left prefrontal cortex region was reduced, and decreased activity in this region may indicate psychomotor inhibition in depression. Thus, standard TMS therapy for depression applies facilitative neuromodulation to the left DLPFC.

This study has several limitations. First, the small sample size used to create an AI model for determining depression from EEG data is a notable limitation. In the present study, we sought to create a model with a minimum sample size by featuring neurophysiological indices closely related to the pathophysiology of depression. Furthermore, we made every effort to increase the reliability of the model by conducting robust internal validations, such as the outer loop of five-fold double cross-validation, whenever possible. However, in the future, it will be necessary to conduct external validation in collaboration with other institutions conducting TMS-EEG studies. Second, we developed an AI algorithm for determining depression using the EEG data from 60 HCs and 60 patients with MDD. There was no significant difference in sex distribution between the two groups, but we were unable to fully match the two groups based on age. As such, we performed statistical analysis by controlling for age post hoc when comparing the mean permutation importance variables between the two groups. Third, the possibility of potential overfitting cannot be ruled out because the present study used a relatively large number of features to create the AI model for a sample size of 120 subjects. Thus, it will be necessary to further increase the sample size to build a more appropriate AI model and to externally validate the accuracy of the present model using a different replication cohort. Fourth, inherent limitations of psychiatry must be considered. The current diagnostic system of psychiatry is based on the operational diagnosis derived from specific symptomatology, which can overlook the detailed symptoms of the patient and force us to provide a specific diagnosis. As such, even patients with the same diagnostic label of “depression” may differ not only in their presenting manifestations but also in the underlying pathophysiology, which can greatly vary from patient to patient. This variance is the cause of the high heterogeneity of the pathological bases as well as of the diversity of symptom phenotypes of depression and other psychiatric disorders. Especially when conducting supervised learning with diagnostic labels, as in the present study, such an approach can inadvertently perpetuate these inherent issues in psychiatry, leading to a tautological trap.

However, the problem of heterogeneity in depression is directly related to the limits of diagnostic accuracy due to the limitations of human intelligence. Through the construction of this depression-determining AI model, we can think using two approaches to understanding the heterogeneity of depression. The first one is to acknowledge the limitations of human intelligence in a negative sense and to take the stance that the diagnosis of depression, which is highly heterogeneous, is fundamentally impossible for humans. Another way of thinking is to recognize the limitations of human intelligence in a positive sense and to actively use the depression-detecting AI model that was developed in this study in real-world psychiatric practice. The AI-determined diagnosis can be compared with each psychiatrist’s own derived diagnostic label each time, and the diagnostic label determined by human psychiatrists can be fed back to the AI algorithm, thereby allowing it to evolve into a more human-oriented and more adaptive AI model. The authors take the latter viewpoint.

Moving forward, it is necessary to develop and implement diagnostic AI algorithms that not only differentiate between health or depression, but also provide more accurate, probable, and useful diagnostic assistance among multiple diagnostic candidates, including bipolar disorder, autism spectrum disorder, schizophrenia, and others.

## 5. Conclusions

In this study, the best estimation performance was achieved when we applied LDA as a machine learning model to the combinations of all four feature sets: pre-stimulus TMS-EEG data, post-stimulus TMS-EEG data, the difference between pre- and post-stimulus TMS-EEG data, and resting-state EEG data. Notably, the present study demonstrated that an AI system for aiding depression determination, boasting a high degree of discriminative accuracy, can be developed by applying an LDA-based machine learning model, with a high degree of dimensionality reduction by featuring the TMS-EEG neurophysiological indices.

## 6. Patents

The following patents have been applied for based on the results of this research (Japanese Patent Application: 2023-098482; United States Application: 18/498,680). 

## Figures and Tables

**Table 1:** Clinico-demographic of the participants in this study.

Characteristic	MDD (n = 60)	HC (n = 60)	Statistics
Age [year]	45.8 ± 12.2	40.5 ± 12.2	t_118_ = −1.89, *p* = 0.06
Female [%]	40	45	χ^2^ = 0.31, *p* = 0.58
Education [year]	15.3 ± 1.9	14.7 ± 2.1	t_118_ = 1.50, *p* = 0.14
MMSE score	29.1 ± 1.4	28.5 ± 3.3	t_118_ = 1.38, *p* = 0.17
Age at onset of TRD [year]	36.0 ± 15.6	—	—
Duration of illness [year]	10.9 ± 9.2	—	—
MADRS score	31.8 ± 7.8	1.1 ± 2.0	t_118_ = −29.40 *p* < 0.001
STAI (trait) score	54.2 ± 20.1	32.1 ± 9.2	t_118_ = −2.88, *p* = 0.005
STAI (state) score	55.4 ± 20.1	34.2 ± 8.9	t_118_ = −2.63, *p* = 0.01

MMSE = Mini-Mental State; STAI = State-Trait Anxiety Inventory; MADRS = Montgomery–Åsberg Depression Rating Scale. — mens N/A

**Table 2:** The mean permutation importance variables appeared in common across all three top machine learning models (LDA, LR, and SVM).

Dataset	Feature	Channel	Band
RST	Power	AFz	β
RST	wPLI	F6-F4	γ
DIF	wPLI	F5-AF3	α
DIF	wPLI	AFz-F5	α
DIF	wPLI	AF3-F8	θ

RST = resting state; DIF = the difference between pre- and post-stimulus TMS-EEG; wPLI = with weighted phase lag index.

## Data Availability

The data presented in this study are available on request from the corresponding author.
